# Bacteriophage-Mediated Modulation of Bacterial Competition during Selective Enrichment of Campylobacter

**DOI:** 10.1128/Spectrum.01703-21

**Published:** 2021-12-15

**Authors:** Jinshil Kim, Jeong In Hur, Sangryeol Ryu, Byeonghwa Jeon

**Affiliations:** a Department of Food and Animal Biotechnology, Research Institute for Agriculture and Life Sciences, Seoul National University, Seoul, Republic of Korea; b Center for Food Bioconvergence, Seoul National University, Seoul, Republic of Korea; c Department of Agricultural Biotechnology, Seoul National University, Seoul, Republic of Korea; d Environmental Health Sciences, School of Public Health, University of Minnesota, Minneapolis, Minnesota, USA; South China Sea Institute of Oceanology, Chinese Academy of Sciences

**Keywords:** *Campylobacter* isolation, bacteriophage, antimicrobial synergy, ESBL-producing *Escherichia coli*

## Abstract

Selective media using antimicrobial supplements generate unique microbial ecology to facilitate bacterial isolation. However, antibiotic-resistant bacteria indigenous to samples can interfere with the isolation process using selective media. Recent studies showed that extended-spectrum beta-lactamase (ESBL)-producing Escherichia coli is highly prevalent on retail raw chicken and compromises the efficacy of Campylobacter isolation because ESBL-producing E. coli are resistant to antimicrobial supplements in Campylobacter-selective media and outgrows Campylobacter. The objective of this study was to improve Campylobacter isolation by inhibiting the growth of ESBL-producing E. coli using bacteriophages (phages). The supplementation of Campylobacter-selective media with E. coli phages reduced the level of ESBL-producing E. coli during the enrichment step. When E. coli phages were combined with the antimicrobial supplements of Campylobacter-selective media, antimicrobial synergy was observed, particularly with rifampicin, an antibiotic used in Preston medium. Although the same materials (i.e., phages and selective media) were used, the sequence of combining the materials markedly influenced the inhibition of ESBL-producing E. coli and the isolation of Campylobacter. These findings indicated that the modulation of microbial competition at the enrichment step was critical to the successful isolation of fastidious bacteria and that phages can be utilized to facilitate the selective enrichment of target bacteria by inhibiting their competitive bacteria.

**IMPORTANCE** Phages are promising antimicrobial alternatives. In this study, we first demonstrated that phages can be used to facilitate selective isolation of fastidious bacteria that are prone to be outgrown by bacterial competitors during isolation. The effectiveness of a phage-based isolation method was primarily dependent on the antimicrobial synergy between phages and antibiotics used in selective media. The same approach could be applied to the development of isolation methods for other fastidious bacteria.

## INTRODUCTION

Bacterial isolation involves a series of procedures that selectively enrich and culture only target bacteria by inhibiting the growth of bacterial competitors that are indigenous to samples. Campylobacter is a bacterial pathogen that contaminates food, particularly retail poultry because Campylobacter heavily inhabits the gastrointestinal tract of avian species (1, 2). However, despite the abundance of Campylobacter in poultry products, it is difficult to isolate Campylobacter mainly because Campylobacter needs fastidious growth requirements. For instance, Campylobacter is unable to use glucose as a nutrient due to a defect in glycolysis and requires microaerobic conditions and elevated temperatures (i.e., 42°C) for optimal growth (3, 4).

Campylobacter isolation is conducted using selective media supplemented with antibiotics, to which Campylobacter is intrinsically resistant (5, 6). Antibiotics in selective supplements are designed to promote the growth of Campylobacter while inhibiting contaminating bacteria. However, the efficacy of Campylobacter isolation has been significantly compromised by bacteria that are resistant to antimicrobial supplements in Campylobacter-selective media. Particularly, extended-spectrum beta-lactamase (ESBL)-producing Escherichia coli are resistant to cefoperazone, a 3rd generation cephalosporin drug used in Campylobacter-selective media, such as Bolton selective medium and modified charcoal-cefoperazone-deoxycholate (mCCD) agar. ESBL-producing E. coli are highly prevalent in chicken and interrupts Campylobacter isolation using current protocols (7, 8), leading to a revision of the international standard for the detection and enumeration of Campylobacter (9, 10). Our previous analysis of microbiome changes during the enrichment of Campylobacter in selective media demonstrated that ESBL-producing E. coli are more abundantly enriched than Campylobacter (11, 12). These studies suggested that inhibiting ESBL-producing E. coli at the enrichment step is the key to the success in Campylobacter isolation.

Because bacteriophages (phages) specifically infect only the host bacteria without affecting other bacteria (13), phages are considered promising antimicrobial alternatives to control antibiotic resistance (14, 15). Unlike antibiotics, phages have unique features, such as strict host specificity, low toxicity, and the capability to self‐replicate (16). However, the use of a single phage is generally ineffective because the host range of phage infection is too narrow, and bacteria resistant to phage infection emerge easily (16). To overcome the limitations, phages are normally used as a cocktail consisting of multiple phages that infect different hosts or recognize different receptors (17). In our recent study, we discovered that E. coli phages have different infection efficacy depending on the phylogenetic group of E. coli (18). Because phylogroups A and B1 are predominant in E. coli isolates from chicken (19–23), a cocktail consisting of phages that infect E. coli in phylogroups A and B1 effectively inhibited antibiotic-resistant E. coli on chicken carcasses (18).

We hypothesized that E. coli phages could be used as an antimicrobial alternative to inhibit ESBL-producing E. coli during the isolation of Campylobacter. Using the E. coli phage cocktail, we demonstrated that phages could be used to facilitate Campylobacter isolation from chicken carcasses by modulating the growth competition between Campylobacter and ESBL-producing E. coli.

## RESULTS

### Phage inhibition of ESBL-producing E. coli in Campylobacter-selective media.

**FIG 1:**
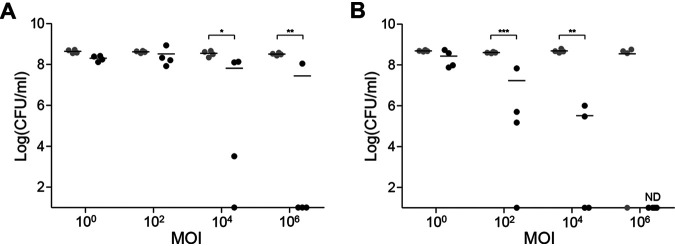
Phage inhibition of ESBL-producing E. coli in Campylobacter-selective media. The inhibition of ESBL-producing E. coli by the phage cocktail in Campylobacter-selective Bolton broth (A) and Campylobacter-selective Preston broth (B). Each dot represents the mean of the results of quadruplicate samples in a single experiment. *, *P* < 0.05; **, *P* < 0.01; ***, *P* < 0.001; ND, not detected (detection limit: 10 CFU/mL); MOI, multiplicity of infection.

Five phages (JEP1, 4, 6, 7, and 8), which effectively inhibit ESBL-producing E. coli isolates from chicken carcasses, were identified and characterized in our previous study (18). Because Campylobacter-selective Bolton broth (BB) and Preston broth (PB) are the culture mediums adopted by the International Organization for Standardization (ISO) in the protocol for Campylobacter isolation (10), BB and PB were used in this study. Although phylogroups A and B1 dominate in E. coli isolates from chicken, E. coli in phylogroups B2 and D have also been isolated from chicken carcasses and can cause extraintestinal infections in humans (19–23). Thus, we prepared a mixed culture consisting of ESBL-producing E. coli strains (E20, E41, E55, and E59), which belong to the phylogroups A, B1, B2, and D, respectively, as reported in our previous study (23). The levels of ESBL-producing E. coli were measured after infecting the mixed culture of E. coli strains with the phage cocktail in BB and PB. Interestingly, the efficacy of E. coli inhibition by the phage cocktail was different depending on the Campylobacter-selective medium (Fig. 1). Whereas the phage cocktail reduced ESBL-producing E. coli marginally (0.7∼1.1 log colony forming units per milliliter [CFU/mL] in mean values) in BB even after treatment with a high multiplicity of infections (MOIs; 10^4^ and 10^6^) (Fig. 1A), the phage cocktail markedly reduced ESBL-producing E. coli in PB in proportion to the MOI (Fig. 1B). At the highest MOI (10^6^), ESBL-producing E. coli were undetectable (<10 CFU/mL) in PB (Fig. 1B). When combined with the phage cocktail, PB generated more significant antimicrobial activity against ESBL-producing E. coli than BB.

### Antimicrobial synergy between phages and antibiotics.

**FIG 2:**
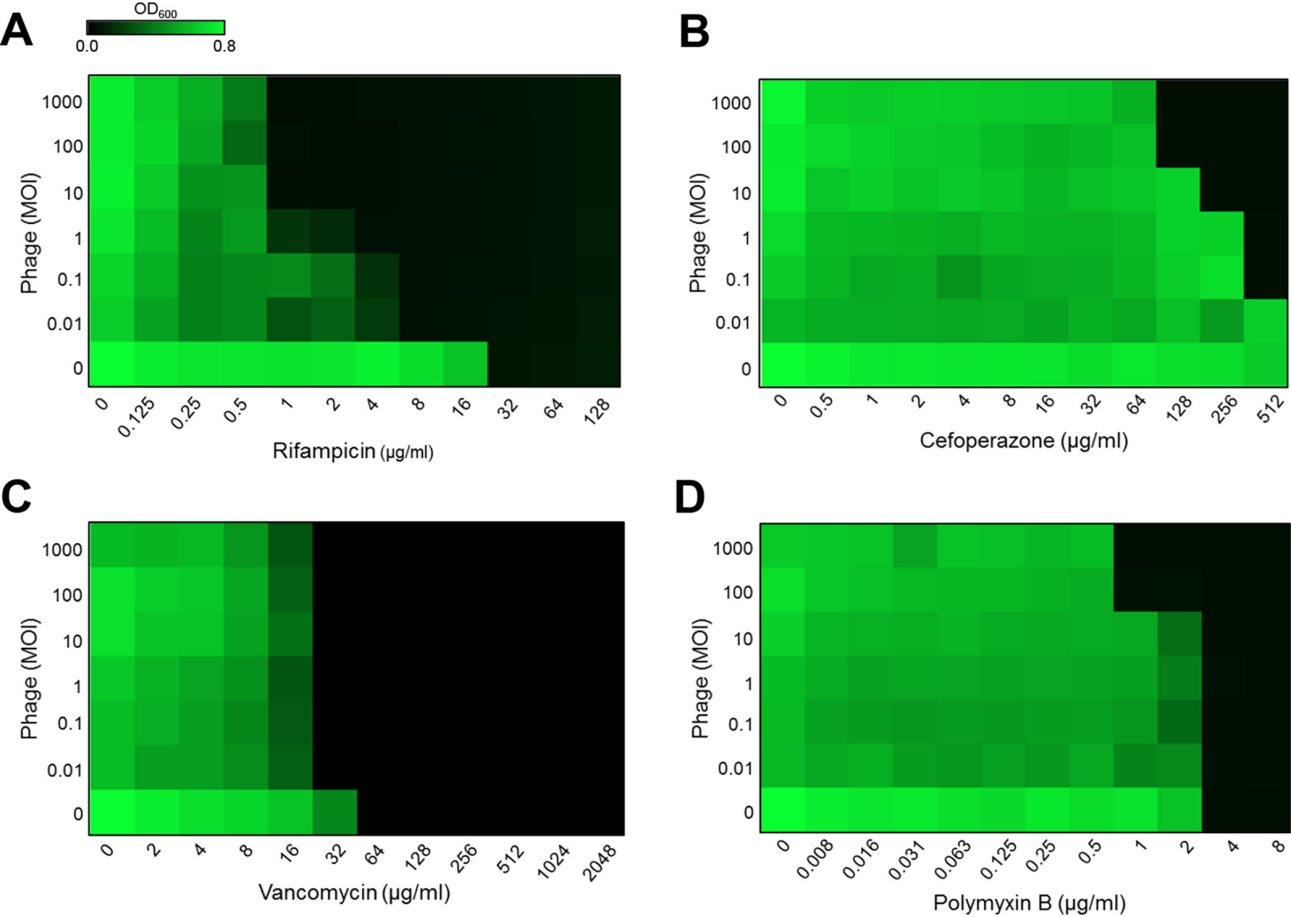
Antimicrobial synergy between phages and antibiotics. Checkerboard titration assays were conducted using the phage cocktail and the antibiotics of Campylobacter-selective Bolton and Preston media, including rifampicin (A), cefoperazone (B), vancomycin (C), and polymyxin B (D). The experiments were repeated at least three times and generated similar results. MOI, multiplicity of infection.

Because the phages inhibited ESBL-producing E. coli differentially depending on the selective medium, we hypothesized that the level of antimicrobial synergy between phages and Campylobacter-selective medium was dependent on the antibiotic used in the selective supplements of BB and PB. The hypothesis was examined by conducting checkerboard titration assays in the presence of five antibiotics (cefoperazone, vancomycin, polymyxin B, rifampicin, and trimethoprim) that are used as antimicrobial supplements in BB and PB. The most substantial synergy was observed when the phage cocktail was combined with rifampicin, an antibiotic supplement in PB (Fig. 2A). Although the MIC of cefoperazone was high (>512 μg/mL) due to cephalosporin resistance that was conferred by ESBL, the susceptibility of ESBL-producing E. coli to cefoperazone was increased when the phages were used at high MOIs (Fig. 2B). The cotreatment with the phage cocktail reduced the MICs of vancomycin and polymyxin B by 2-fold and 4-fold, respectively (Fig. 2C and D). Trimethoprim is commonly used as an antibiotic supplement in both BB and PB. Despite cotreatment with the phage cocktail, trimethoprim did not reduce the level of ESBL-producing E. coli (Fig. S1). These results indicated that phages could enhance the antimicrobial activity of antibiotics when used together, and the efficacy of antimicrobial synergy was affected by the antibiotic combined with phages. Based on these findings, the substantial inhibition of ESBL-producing E. coli by the phage cocktail in PB (Fig. 1B) could be ascribed to the antimicrobial synergy between the phages and rifampicin (Fig. 2A).

### Inhibition of ESBL-producing E. coli by phages during selective enrichment.

**FIG 3:**
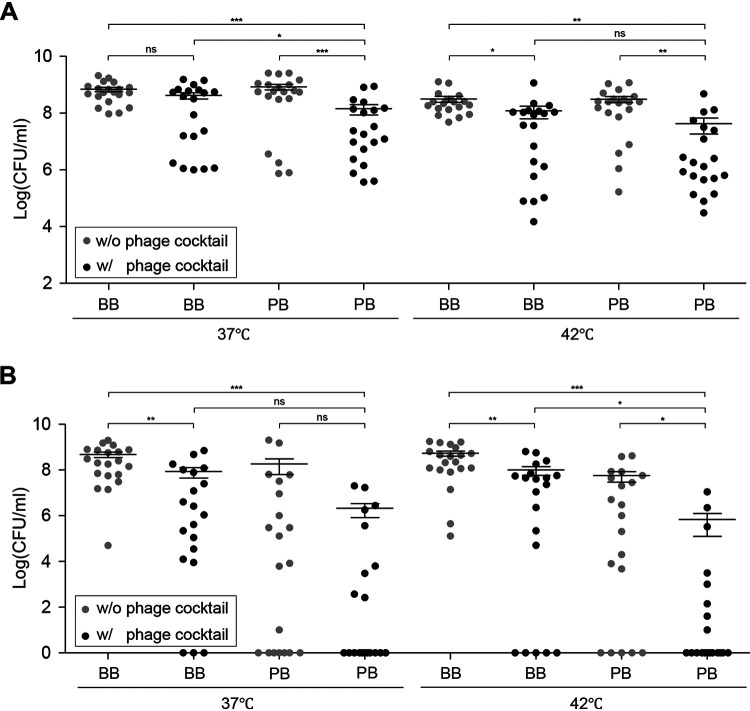
Inhibition of ESBL-producing E. coli by the phage cocktail in Campylobacter-selective enrichment broth. The level of ESBL-producing E. coli was determined in 20 chicken carcasses in Campylobacter-selective enrichment media using qPCR amplifying *uidA* (A) and CFU counting on MacConkey agars supplemented with 1 μg/mL cefotaxime (B). SM buffer was added to nontreated controls. BB, Bolton broth; PB, Preston broth. The bars show the mean and the standard deviations. *, *P* < 0.05; **, *P* < 0.01; ***, *P* < 0.001; ns, not significant.

The levels of ESBL-producing E. coli were measured under eight different enrichment conditions that were made by combining two kinds of selective medium (BB and PB), two temperatures (37°C and 42°C), and with/without the phage cocktail (Fig. S2). After enrichment of chicken samples under the eight enrichment conditions, the levels of E. coli were quantified by qPCR (Fig. 3A). E. coli was detected in all samples, and the phages significantly reduced E. coli under all the conditions except on BB at 37°C (Fig. 3A). Moreover, phage treatment substantially reduced the level of ESBL-producing E. coli based on the enumeration of colonies on MacConkey agars supplemented with cefotaxime, a 3rd generation cephalosporin drug (Fig. 3B). E. coli was significantly reduced when the phages were used in PB at 42°C (Fig. 3). In contrast, the level of Campylobacter was increased by phage treatment under the enrichment conditions, but the differences were not statistically significant (Fig. S3), suggesting unidentified factors may still affect the enrichment of Campylobacter. Nevertheless, these results suggested that the phages successfully reduced the level of ESBL-producing E. coli, the major bacterial competitor affecting the growth of Campylobacter.

### Improvement of Campylobacter isolation by modulating bacterial competition using phages.

**FIG 4:**
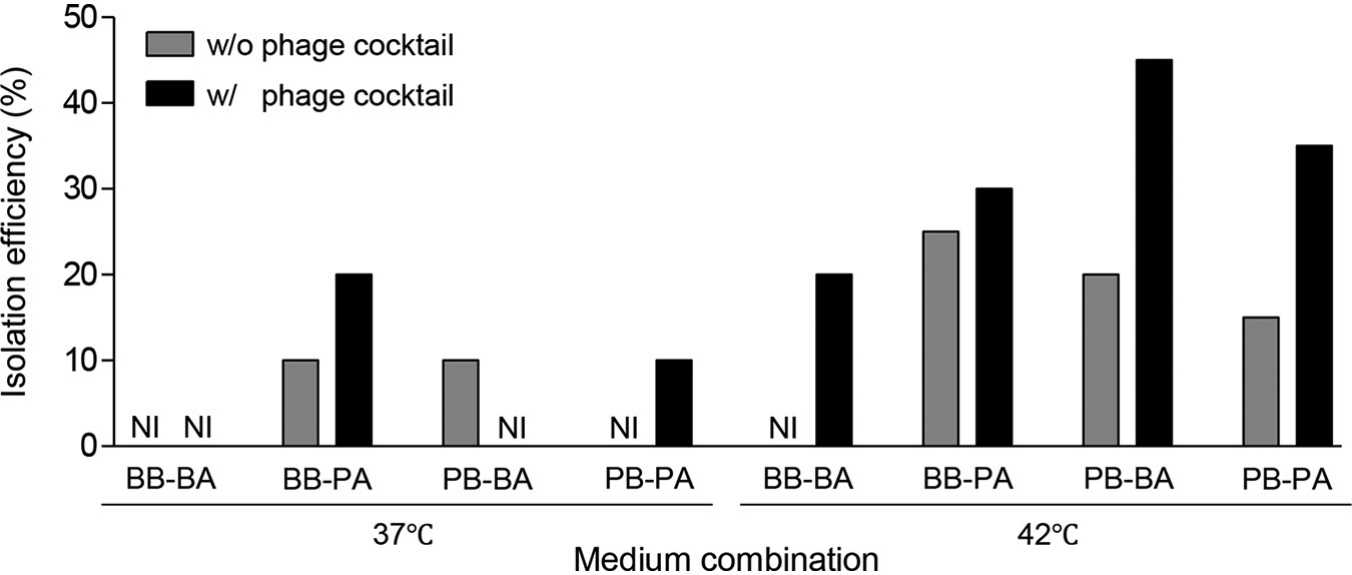
Effects of phage cocktail treatment on Campylobacter isolation. NI, not isolated; BB, Bolton broth; BA, Bolton agar; PB, Preston broth; PA, Preston agar.

To evaluate how phage inhibition of E. coli affects Campylobacter isolation, the frequency of Campylobacter isolation was measured in 16 different culture conditions where we combined two of the 1st enrichment mediums (BB and PB), two of the 2nd selective solid medium (Bolton agar [BA] and Preston agar [PA]), two enrichment temperatures (37°C and 42°C), and with/without the phage cocktail (Fig. S2). Depending on the condition, the isolation frequencies varied from 0% to 45% (Fig. 4 and Table S2). Campylobacter was not isolated in 8 out of 20 chicken samples under the 16 conditions (Table S2). The use of the phage cocktail at the enrichment step markedly increased the isolation frequency, especially when the samples were enriched at 42°C in PB followed by plating on BA (Fig. 4).

**FIG 5:**
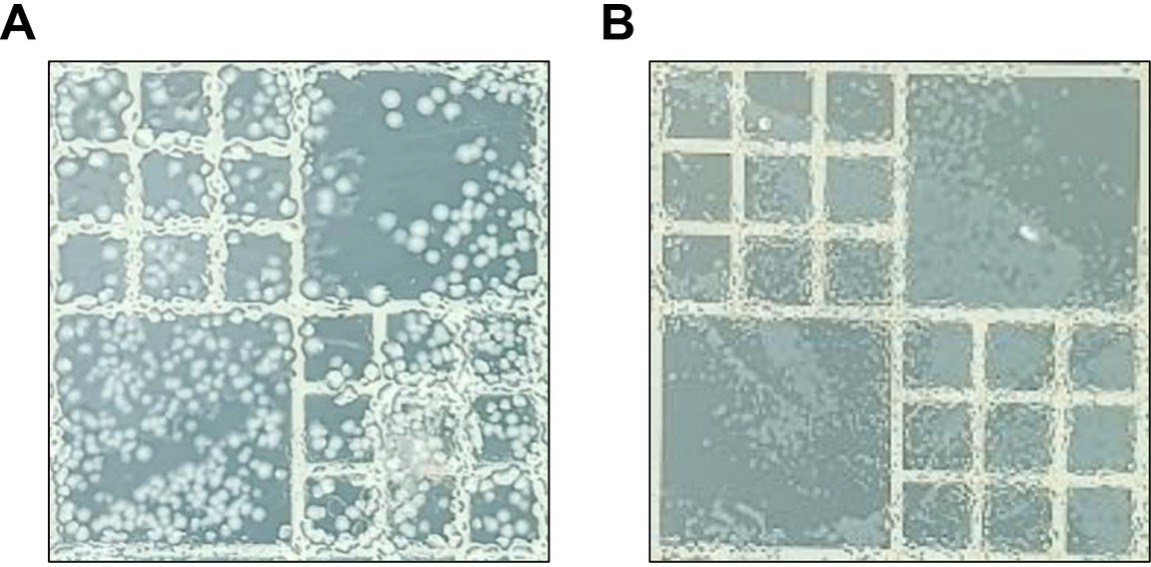
Reduced formation of ESBL-producing E. coli colonies by phage treatment. The photos show representative Bolton agars inoculated with an aliquot of Campylobacter-selective Preston enrichment broth without (A) and with (B) the phage cocktail at 42°C. Whitish colonies are ESBL-producing E. coli and transparent colonies are Campylobacter.

Moreover, when the phage cocktail was added to the enrichment medium, the number of E. coli colonies was substantially reduced, whereas Campylobacter colonies were easily recognized (Fig. 5). Without using the phage cocktail, Campylobacter-selective agar plates were heavily covered with colonies of ESBL-producing E. coli (Fig. 5A). However, the use of the phage cocktail markedly prevented the formation of E. coli colonies and made Campylobacter colonies easily recognizable (Fig. 5B), indicating that the phages successfully inhibited ESBL-producing E. coli and facilitated the growth of Campylobacter.

## DISCUSSION

Successful bacterial isolation relies on how effectively selective culture media can eliminate bacterial competitors while facilitating the growth of the target bacteria. Our previous studies demonstrated that each Campylobacter-selective medium makes substantial changes in the microbiome of enrichment cultures because of the selective pressure generated by antimicrobial supplements (11, 12). Proteobacteria and Firmicutes are the major phyla after the enrichment of chicken carcasses with BB, whereas Proteobacteria and Fusobacteria were dominant after the enrichment with PB (11). Commonly, antibiotic-resistant E. coli, such as ESBL-producing E. coli, is predominantly enriched in both BB and PB (11). Consistently, it has been reported that ESBL-producing E. coli prevalent on chicken carcasses significantly compromises the efficacy of Campylobacter isolation (7, 11), suggesting the need for improving the current isolation methods. However, ESBL genes in E. coli are generally encoded on plasmids harboring a cluster of antibiotic resistance genes (24, 25). Thus, the identification of additional antimicrobial supplements, which inhibit ESBL-producing E. coli and do not affect the growth of Campylobacter, is extremely challenging.

**FIG 6:**
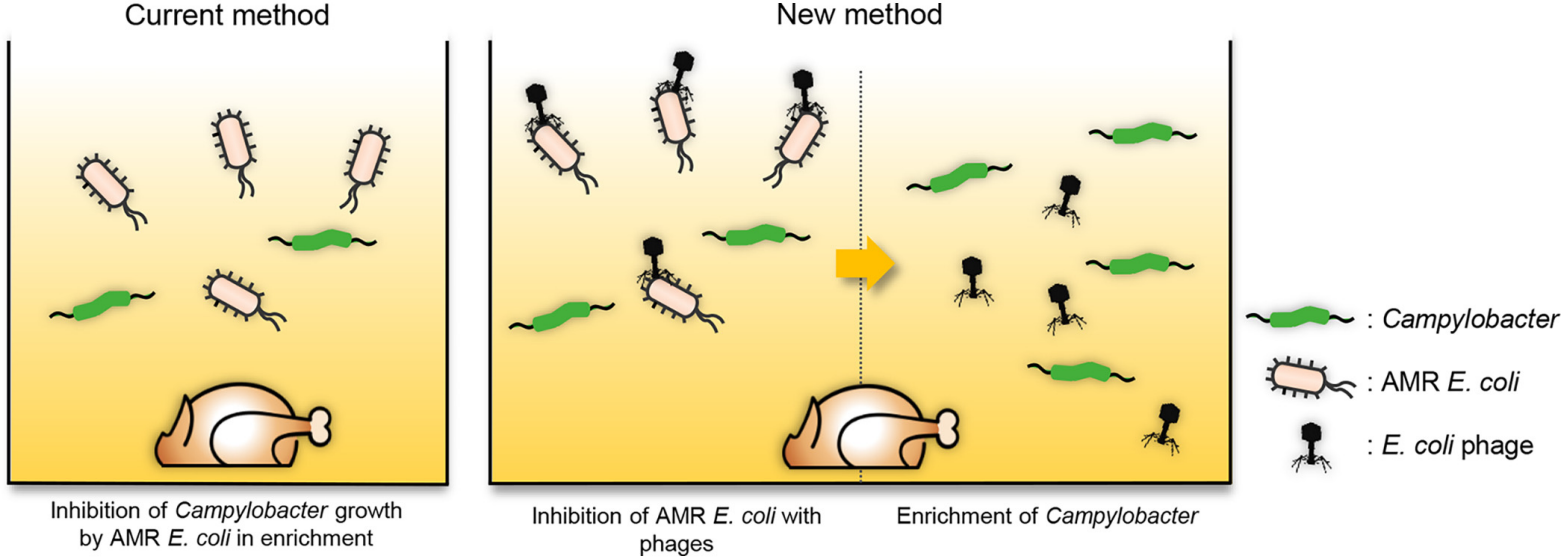
Modulation of microbial competition with phages at the step of Campylobacter enrichment. AMR, antibiotic-resistant.

To address this issue, we used phages as an antimicrobial supplement to inhibit ESBL-producing E. coli, the primary bacterial competitor interrupting Campylobacter isolation. The isolation of fastidious bacteria, such as Campylobacter, is a technical challenge to many microbiology laboratories because Campylobacter is normally outcompeted by other fast-growing bacteria that are indigenous to samples. For this, selective media were supplemented with antibiotics to enhance the isolation of Campylobacter by suppressing competing bacteria (11, 26). This study first demonstrated that phages can be utilized to improve the efficacy of Campylobacter isolation by controlling microbial competition between Campylobacter and ESBL-producing E. coli (Fig. 6). When current methods are used to isolate Campylobacter from chicken, ESBL-producing E. coli could outgrow Campylobacter during enrichment (Fig. 6). However, phages could inhibit ESBL-producing E. coli and improved Campylobacter isolation (Fig. 6).

The antimicrobial synergy between phages and antibiotics enhanced the inhibition of ESBL-producing E. coli, indicating that phages could be used complementarily with antibiotics to inhibit antibiotic-resistant bacteria. Importantly, the level of antimicrobial synergy between phages and antibiotics was affected by the antibiotic combined with phages. The most significant antimicrobial synergy was observed when the phages were mixed with rifampicin, a broad-spectrum antibiotic used in PB. The phage cocktail reduced the MIC of rifampicin by 32-fold even at low MOIs, such as 10 (Fig. 2A). The synergy between phages and rifampicin may account for the substantial reduction in ESBL-producing E. coli by the phage cocktail in PB (Fig. 1B). In addition, the level of antimicrobial susceptibility is another factor that determines antimicrobial synergy. The phage treatment reduced the MIC only by 2-fold in combination with vancomycin regardless of MOIs, because vancomycin is not effective against Gram-negative bacteria. A recent study also reported that phages generate synergistic antimicrobial effects with antibiotics depending on the mode of action of an antibiotic possibly because the impairment of critical cellular functions in bacteria, such as DNA replication and protein synthesis, by antibiotics can also affect the life cycle of phages within bacterial hosts (27).

It is worth noting that the sequence of combining selective mediums made substantial differences in the inhibition of ESBL-producing E. coli and, consequently, the efficacy of Campylobacter isolation. Although the same selective medium and phages were used in the experiments, the frequency of Campylobacter isolation varied widely depending on how the materials were used in combination (Fig. 4). The highest isolation efficiency was achieved when chicken samples were enriched in PB in the presence of the phage cocktail at 42°C followed by plating on BA (Fig. 4). However, the enrichment of chicken samples in BB with the phage cocktail followed by plating on PA exhibited only 20% of isolation efficiency at 37°C (Fig. 4). Moreover, enrichment temperatures were another important factor determining the efficacy of Campylobacter isolation using phages. Overall, the frequency of Campylobacter isolation was higher at 42°C than 37°C (Fig. 4), presumably because Campylobacter optimally grows at 42°C. Although the supplementation of selective media with phages provides additional selective pressure to improve Campylobacter isolation, environmental factors should also be optimized to facilitate the growth of target bacteria.

Here, we demonstrated that phages can be used to facilitate the isolation of Campylobacter from retail raw chicken by inhibiting ESBL-producing E. coli, the primary bacterial competitor interrupting Campylobacter isolation. The findings in this study provide new insights into how phages can be incorporated into the procedures to isolate fastidious bacteria.

## MATERIALS AND METHODS

### Bacterial strains, phages, and growth conditions.

Five phages (JEP1, 4, 6, 7, and 8) were reported in our previous study to inhibit ESBL-producing E. coli isolates from chicken carcasses (18). All cesium chloride (CsCl)-purified phages (≥10^10^ plaque forming units per milliliter [PFU/mL]) were used in this experiment. ESBL-producing E. coli strains E20, E41, E55, and E59 belonging to phylogroups A, B1, B2, and D, respectively, were isolated from retail raw chicken in our previous study (18) and routinely cultured at 37°C in Luria Bertani (LB) medium (Difco, NJ, USA). Campylobacter was cultured at 42°C in Mueller-Hinton (MH) medium (Oxoid, Hampshire, UK) under microaerobic conditions (5% O_2_, 10% CO_2_, 85% N_2_). All bacterial strains were stored at −80°C in LB or MH broth with 15% glycerol. The sodium chloride-magnesium sulfate (SM) buffer (100 mM NaCl, 8 mM MgSO_4_·7H_2_O, and 50 mM Tris·HCl, pH 7.5) was used as the phage buffer.

### Inhibition assay in Bolton and Preston Campylobacter-selective mediums.

Overnight cultures of ESBL-producing E. coli strains E20, E41, E55, and E59 in LB broth were diluted in fresh LB broth by 100-fold. After the cultivation of the ESBL-producing E. coli strains (E20, E41, E55, and E59) to an optical density at 600 nm (OD_600_) of 0.5, an equal volume of the cultures was mixed in a single tube. The mixed culture was diluted and added to 3 mL of Campylobacter-selective Bolton medium (Oxoid, UK) and Preston medium (Thermo-Fisher Scientific, OH, USA) at 10^1^, 10^3^, 10^5^, or 10^7^ CFU/mL followed by the addition of the phage cocktail (40 μL; 10^7^ PFU/mL) at a range of MOI values (10° to 10^6^). After incubation at 42°C for 24 h, a 10-fold serially diluted culture was plated onto LB agar plates to enumerate E. coli.

### Checkerboard titration assay.

The checkerboard titration assay was performed as described previously (28) to evaluate whether phages could inhibit ESBL-producing E. coli synergistically with the antibiotics in Bolton and Preston mediums, including cefoperazone (0 to 512 μg/mL; MilliporeSigma, MO, USA), vancomycin (0 to 2048 μg/mL; MilliporeSigma), and trimethoprim (0 to 512 μg/mL; MilliporeSigma) in Bolton medium, and polymyxin B (0 to 8 μg/mL; MilliporeSigma), rifampicin (0 to 128 μg/mL; MilliporeSigma), and trimethoprim (0 to 512 μg/mL; MilliporeSigma) in Preston medium. Five antibiotics (cefoperazone, vancomycin, polymyxin B, rifampicin, and trimethoprim) were selected for the assay. The antibiotics were serially diluted 2-fold on each column in a 96-well plate, and the phage cocktail was diluted 10-fold on each row. The mixed culture of E. coli strains (E20, E41, E55, and E59) was prepared as described above and added to the 96-well plate (10^5^ CFU per well). The plate was incubated at 42°C for 24 h. The OD_600_ was measured with SpectaMax i3 Platform (Molecular Devices, CA, USA).

### Sample collection and selective enrichment.

Twenty whole chicken carcasses (20 different brands of 16 different companies) were purchased from retail stores. The thigh meat was cut with a sterilized knife. Approximately 10 g of chicken thigh meat was enriched with 90 mL of Bolton or Preston broth with phage cocktail (10^8^ PFU/mL) or SM buffer at different temperatures (37°C or 42°C) for 24 h under microaerobic conditions.

### Quantitative PCR (qPCR) of E. coli in enrichment broth.

To determine the level of E. coli at the enrichment step, qPCR was performed as described previously (29). A standard curve was prepared using the genomic DNA (gDNA) of E. coli K-12 W3110. The gDNA was extracted from E. coli K-12 W3110 grown to an OD_600_ of 1 using the G-spin™ Genomic DNA Extraction kit (Intronbio, Seongnam, Republic of Korea) according to the manufacturer’s instructions. The purified gDNA was serially diluted to create a standard curve. A standard curve correlating gDNA copy number with CFU was generated using *uidA* (29). The enrichment cultures (20 mL) were concentrated by centrifugation at 4,000 × *g*, 4°C for 7 min, and pellets were resuspended with 2 mL of PBS. The gDNA was extracted from 1 mL resuspension and analyzed by qPCR. The qPCR mixture contained 10 μL 2 × iQ SYBR green Supermix (Bio-Rad, CA, USA) and 0.3 μM each primer in a reaction volume of 20 μL, uidA-qPCR-F, and uidA-qPCR-R (Table S1). All qPCRs were performed using the CFX Connect™ real-time PCR detection system (Bio-Rad, USA), and the cycling parameters were as follows: 95°C for 5 min; 39 cycles at 95°C for 15 s, 55°C for 15 s, 72°C for 30 s; 72°C for 7 min.

### Quantification of ESBL-producing E. coli in enrichment broth.

Enrichment cultures (20 mL) were centrifuged at 4,000 × *g*, 4°C for 7 min and resuspended with 2 mL of PBS. ESBL-producing E. coli was counted by 10-fold serial dilution and plating on MacConkey agars (Oxoid, UK) supplemented with 1 μg/mL cefotaxime.

### Isolation of Campylobacter under 16 different combinations.

Enrichment cultures (100 μL) prepared as described above were spread onto Bolton and Preston agar plates supplemented with Campylobacter-selective supplements. The plates were incubated at 42°C for 48 h under microaerobic conditions. For each sample, 15 colonies were analyzed and isolated to indicate Campylobacter isolation efficiency. Based on colony morphology, 15 presumptive Campylobacter colonies were randomly selected and tested with multiplex PCR using the primer sets specific for Campylobacter 16S rRNA and three Campylobacter species, including Campylobacter jejuni, Campylobacter coli, and Campylobacter lari (Table S1).

### Statistics analysis.

Statistical significance was evaluated with Student's *t* test using GraphPad Prism version 5.01 (GraphPad Software, Inc., CA, USA).
