# Supplementary material for: Bacteriophage-Mediated Modulation of Bacterial Competition during Selective Enrichment of Campylobacter
Source: Microbiol Spectr. 2021 Dec 15;9(3):e01703-21. doi: 10.1128/Spectrum.01703-21 (PMC8672905; doi:10.1128/Spectrum.01703-21)
Supplement: SUPPLEMENTAL FILE 1 — Supplemental material. Download SPECTRUM01703-21_Supp_1_seq10.pdf, PDF file, 0.4 MB [file spectrum01703-21_supp_1_seq10.pdf]

Table S1. Primers used in this study

Genus	Gene	Primer	Sequence (5′-3′)	Reference
Escherichia coli	*uidA*	uidA-qPCR-F	AGGTGGTTGCAACTGGACAA	This study
		uidA-qPCR-R	TCAGGAACTGTTCGCCCTTC	
Campylobacter spp.	16s rRNA	16s-qPCR-F	ATAAGCACCGGCTAACTCCG	This study
		16s-qPCR-R	TTCCATCTGCCTCTCCCTCA	
	16s rRNA	C412F	GGATGACACTTTTCGGAGC	(1)
		C1228R	CATTGTAGCACGTGTGTC	
	cj0414	C-1	CAAATAAAGTTAGAGGTAGAATGT	(2)
		C-3	CCATAAGCACTAGCTAGCTGAT	
	hipO	hipO-F	GCAAAATCCACAGCTTCATCGT	(3)
		hipO-R	GGAAGGGGTGGTCATGGAAG	
	ask	CC18F	GGTATGATTTCTACAAAGCGAG	(1)
		CC519R	ATAAAAGACTATCGTCGCGTG	

Table S2. Effects of phage treatment on *Campylobacter* isolation from chicken carcasses[a]

Enrichment Conditions	Temperature	37°C								42°C							
	**Selective media**	**Bolton**				**Preston**				**Bolton**				**Preston**			
	**Phage[b]**	**N**		**Y**		**N**		**Y**		**N**		**Y**		**N**		**Y**	
**2nd Selection**	**Selective media**	**Bolton**	**Preston**	**Bolton**	**Preston**	**Bolton**	**Preston**	**Bolton**	**Preston**	**Bolton**	**Preston**	**Bolton**	**Preston**	**Bolton**	**Preston**	**Bolton**	**Preston**
**Sample no.**	**#1**	0/15	0/15	0/15	0/15	0/15	0/15	0/15	0/15	0/15	0/15	0/15	0/15	**2/15**	0/15	**15/15**	**11/15**
	**#2**	0/15	0/15	0/15	0/15	0/15	0/15	0/15	0/15	0/15	0/15	0/15	0/15	0/15	0/15	0/15	0/15
	**#3**	0/15	0/0	0/15	**2/15**	0/15	0/15	0/0	**1/15**	0/15	0/15	0/15	**14/15**	0/15	**6/15**	**15/15**	0/15
	**#4**	0/15	0/15	0/15	0/0	0/15	0/15	0/3	0/15	0/15	0/15	0/15	0/0	0/15	0/15	0/0	0/15
	**#5**	0/15	0/0	0/15	0/15	0/15	0/15	0/15	**3/15**	0/15	0/15	0/15	0/0	0/15	0/15	0/15	**2/15**
	**#6**	0/15	0/7	0/15	**14/15**	**8/15**	0/15	0/15	0/15	0/15	**15/15**	0/15	**15/15**	**8/15**	**3/15**	**15/15**	**15/15**
	**#7**	0/15	0/0	0/0	0/0	**1/15**	0/15	0/15	0/15	0/15	0/3	**12/15**	0/15	**15/15**	0/15	**15/15**	0/15
	**#8**	0/15	**1/15**	0/15	**15/15**	0/15	0/15	0/0	0/0	0/15	**15/15**	**15/15**	**15/15**	0/15	0/15	**4/15**	**9/15**
	**#9**	0/15	0/15	0/15	0/15	0/15	0/15	0/15	0/15	0/15	0/15	0/15	0/15	0/15	0/15	0/15	0/15
	**#10**	0/15	0/15	0/15	0/0	0/0	0/15	0/0	0/0	0/15	0/15	0/15	0/0	0/0	0/3	0/0	0/15
	**#11**	0/15	0/15	0/15	0/15	0/15	0/15	0/15	0/15	0/15	0/15	0/15	**15/15**	0/15	**1/15**	0/15	0/15
	**#12**	0/15	0/15	0/15	0/15	0/15	0/15	0/15	0/15	0/15	0/15	0/15	0/15	0/15	0/15	**15/15**	0/15
	**#13**	0/15	0/15	0/15	0/13	0/0	0/15	0/0	0/15	0/15	0/15	0/8	0/15	0/15	0/15	0/15	0/15
	**#14**	0/15	0/15	0/0	0/0	0/15	0/15	0/15	0/15	0/15	**10/15**	0/15	0/15	0/15	0/15	0/15	**15/15**
	**#15**	0/15	0/15	0/15	0/15	0/15	0/15	0/15	0/15	0/15	**10/15**	**13/15**	**15/15**	0/15	0/15	**15/15**	**1/15**
	**#16**	0/15	**2/2**	0/15	**5/5**	0/15	0/15	0/15	0/15	0/15	**15/15**	**15/15**	**15/15**	**7/15**	0/15	**15/15**	**4/15**
	**#17**	0/15	0/0	0/15	0/15	0/15	0/15	0/0	0/0	0/15	0/0	0/1	0/0	0/15	0/15	0/0	0/15
	**#18**	0/15	0/0	0/5	0/15	0/15	0/15	0/0	0/15	0/15	0/1	0/15	0/15	0/15	0/15	0/15	0/15
	**#19**	0/15	0/0	0/5	0/15	0/15	0/15	0/0	0/15	0/15	0/1	0/15	0/15	0/15	0/15	0/15	0/15
	**#20**	0/15	0/15	0/15	0/15	0/15	0/15	0/15	0/15	0/15	0/15	0/15	0/0	0/15	0/15	**7/15**	0/15
	**Frequency**	**0%**	**10%**	**0%**	**20%**	**10%**	**0%**	**0%**	**10%**	**0%**	**25%**	**20%**	**30%**	**20%**	**15%**	**45%**	**35%**

[a] no. of *Campylobacter* positive colonies / no. of tested colonies.
[b] N: without phage cocktail, Y: with phage cocktail.

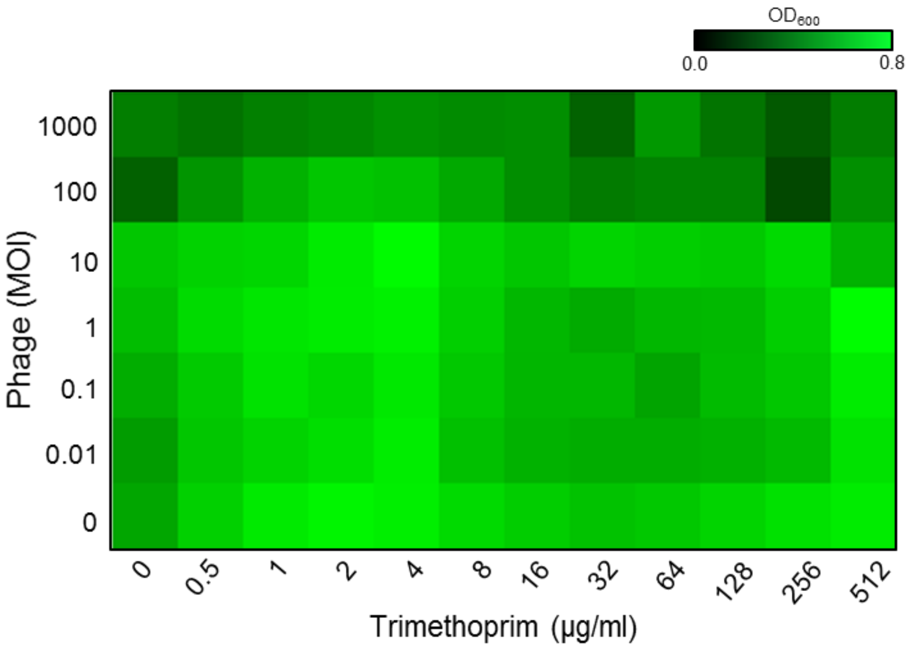

Fig. S1. Growth of ESBL-producing *E. coli* in the presence of phages and trimethoprim. Checkerboard titration assay was conducted using the phage cocktail and trimethoprim, an antibiotic commonly used in both Bolton and Preston *Campylobacter*-selective media. The combinational treatment with phages and trimethoprim did not generate any synergistic antimicrobial activity.

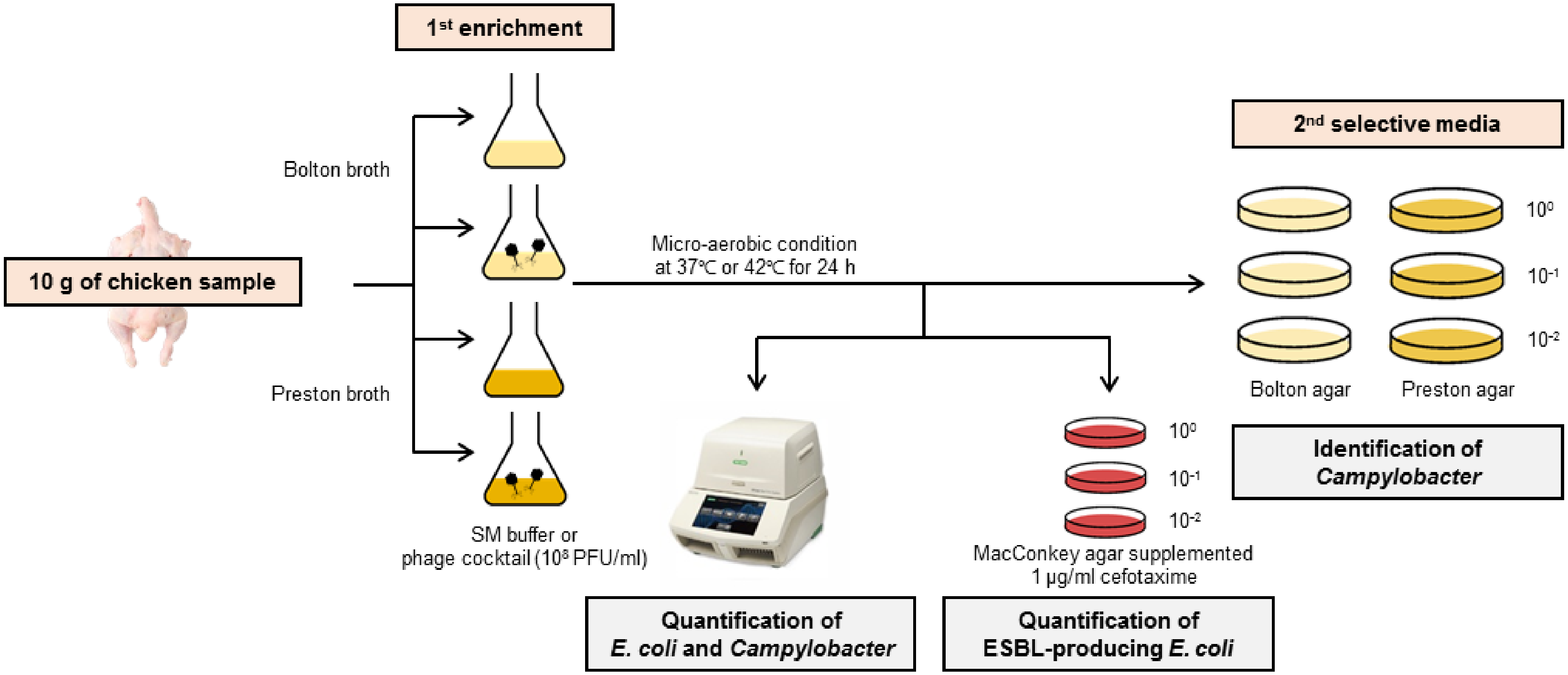

Fig. S2. Experimental procedures to determine the levels of *E. coli* and *Campylobacter* after treatment with phages.

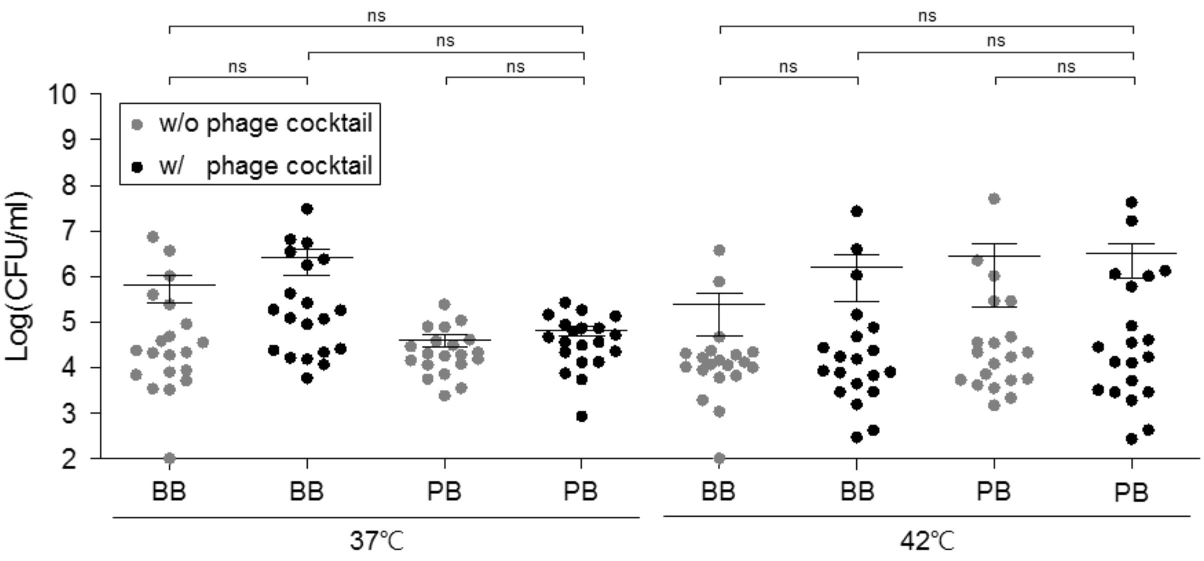

Fig. S3. The levels of *Campylobacter* in enrichment cultures. *Campylobacter* quantification in each combination was obtained by qPCR using 16s rRNA gene (Table S1). SM buffer was added to enrichment broth as a negative control. Statistical analysis was performed using GraphPad Prism, and significance was tested by Student's *t* test. ns, not significant.

References

1. Linton D, Owen R, Stanley J. 1996. Rapid identification by PCR of the genus *Campylobacter* and of five *Campylobacter* species enteropathogenic for man and animals. Res Microbiol 147:707-718.
2. Wang R-f, Slavik MF, Cao W-w. 1992. A rapid PCR method for direct detection of low numbers of *Campylobacter jejuni*. J Rapid Meth Aut Mic 1:101-108.
3. Kim J, Park H, Kim J, Kim JH, Jung JI, Cho S, Ryu S, Jeon B. 2019. Comparative analysis of aerotolerance, antibiotic resistance, and virulence gene prevalence in *Campylobacter jejuni* isolates from retail raw chicken and duck meat in South Korea. Microorganisms 7:433.
